# Supplementary material for: Bayesian refinement of protein structures and ensembles against SAXS data using molecular dynamics
Source: PLoS Comput Biol. 2017 Oct 18;13(10):e1005800. doi: 10.1371/journal.pcbi.1005800 (PMC5662244; doi:10.1371/journal.pcbi.1005800)
Supplement: S1 Archive — (BZ2) [file pcbi.1005800.s011.tar.bz2 › gromacs_4.62_bayesian_saxs_refi/share/html/online/gmxcheck.html]

gmxcheck


| gmxcheck Main Table of Contents |

**VERSION 4.6  
Sat 19 Jan 2013**

---

### Description

gmxcheck reads a trajectory (.trj, .trr or
.xtc), an energy file (.ene or .edr)
or an index file (.ndx)
and prints out useful information about them.

Option -c checks for presence of coordinates,
velocities and box in the file, for close contacts (smaller than
-vdwfac and not bonded, i.e. not between -bonlo
and -bonhi, all relative to the sum of both Van der Waals
radii) and atoms outside the box (these may occur often and are
no problem). If velocities are present, an estimated temperature
will be calculated from them.

If an index file, is given its contents will be summarized.

If both a trajectory and a .tpr file are given (with -s1)
the program will check whether the bond lengths defined in the tpr
file are indeed correct in the trajectory. If not you may have
non-matching files due to e.g. deshuffling or due to problems with
virtual sites. With these flags, gmxcheck provides a quick check for such problems.

The program can compare two run input (.tpr, .tpb or
.tpa) files
when both -s1 and -s2 are supplied.
Similarly a pair of trajectory files can be compared (using the -f2
option), or a pair of energy files (using the -e2 option).

For free energy simulations the A and B state topology from one
run input file can be compared with options -s1 and -ab.

In case the -m flag is given a LaTeX file will be written
consisting of a rough outline for a methods section for a paper.

### Files

| option | filename | type | description |
| --- | --- | --- | --- |
| **-f** | traj.xtc | Input, Opt. | Trajectory: xtc trr trj gro g96 pdb cpt |
| **-f2** | traj.xtc | Input, Opt. | Trajectory: xtc trr trj gro g96 pdb cpt |
| **-s1** | top1.tpr | Input, Opt. | Run input file: tpr tpb tpa |
| **-s2** | top2.tpr | Input, Opt. | Run input file: tpr tpb tpa |
| **-c** | topol.tpr | Input, Opt. | Structure+mass(db): tpr tpb tpa gro g96 pdb |
| **-e** | ener.edr | Input, Opt. | Energy file |
| **-e2** | ener2.edr | Input, Opt. | Energy file |
| **-n** | index.ndx | Input, Opt. | Index file |
| **-m** | doc.tex | Output, Opt. | LaTeX file |

### Other options

| option | type | default | description |
| --- | --- | --- | --- |
| **-[no]h** | bool | no | Print help info and quit || **-[no]version** | bool | no | Print version info and quit || **-nice** | int | 0 | Set the nicelevel || **-vdwfac** | real | 0.8 | Fraction of sum of VdW radii used as warning cutoff || **-bonlo** | real | 0.4 | Min. fract. of sum of VdW radii for bonded atoms || **-bonhi** | real | 0.7 | Max. fract. of sum of VdW radii for bonded atoms || **-[no]rmsd** | bool | no | Print RMSD for x, v and f || **-tol** | real | 0.001 | Relative tolerance for comparing real values defined as 2\*(a-b)/(\|a\|+\|b\|) || **-abstol** | real | 0.001 | Absolute tolerance, useful when sums are close to zero. || **-[no]ab** | bool | no | Compare the A and B topology from one file || **-lastener** | string |  | Last energy term to compare (if not given all are tested). It makes sense to go up until the Pressure. |

---

http://www.gromacs.org  
gromacs@gromacs.org
